# Understanding Cortical Streams from a Computational Perspective

**DOI:** 10.1162/jocn_a_02121

**Published:** 2024-12-01

**Authors:** Zhixian Han, Anne B. Sereno

**Affiliations:** Purdue University; Indiana University School of Medicine

## Abstract

The two visual cortical streams hypothesis, which suggests object properties (what) are processed separately from spatial properties (where), has a longstanding history, and much evidence has accumulated to support its conjectures. Nevertheless, in the last few decades, conflicting evidence has mounted that demands some explanation and modification. For example, existence of (1) shape activities (fMRI) or shape selectivities (physiology) in dorsal stream, similar to ventral stream; likewise, spatial activations (fMRI) or spatial selectivities (physiology) in ventral stream, similar to dorsal stream; (2) multiple segregated subpathways within a stream. In addition, the idea of segregation of various aspects of multiple objects in a scene raises questions about how these properties of multiple objects are then properly re-associated or bound back together to accurately perceive, remember, or make decisions. We will briefly review the history of the two-stream hypothesis, discuss competing accounts that challenge current thinking, and propose ideas on why the brain has segregated pathways. We will present ideas based on our own data using artificial neural networks (1) to reveal encoding differences for what and where that arise in a two-pathway neural network, (2) to show how these encoding differences can clarify previous conflicting findings, and (3) to elucidate the computational advantages of segregated pathways. Furthermore, we will discuss whether neural networks need to have multiple subpathways for different visual attributes. We will also discuss the binding problem (how to correctly associate the different attributes of each object together when there are multiple objects each with multiple attributes in a scene) and possible solutions to the binding problem. Finally, we will briefly discuss problems and limitations with existing models and potential fruitful future directions.

## HISTORY

As early as the late 19th century, researchers had shown that primates with parietal lesions displayed difficulty with localizing objects (Ferrier & Yeo, 1884) and those with temporal lesions had difficulty discriminating objects (Brown & Sharpey-Schafer, 1888). By 1982, Ungerleider and Mishkin (1982) had added their own findings as well as reviewed a rich literature of lesion, neuropsychological, and anatomical studies that supported the hypothesis that there are two distinct cortical pathways in the human visual system (Felleman & Van Essen, 1991; Mishkin, Ungerleider, & Macko, 1983). In addition, Ungerleider, Courtney, and Haxby (1998) suggested that the segregation of ventral and dorsal visual streams extends forward into the human pFC. Furthermore, studies conducted by Courtney, Ungerleider, Keil, and Haxby (1996) showed that the ventrolateral areas of pFC were more active when processing working memory of faces whereas the dorsolateral areas of pFC were more active when processing working memory of spatial locations. More recent reviews of perceptual and visuomotor deficits observed in children (Goodale, 2013) and even effective connectivity (Rolls, Deco, Huang, & Feng, 2023) continue to support the idea of a ventral pathway specialized for processing information about object recognition (Logothetis & Sheinberg, 1996), and a dorsal pathway specialized for processing information about visuospatial cognition (Colby & Goldberg, 1999).

However, in the last few decades, other studies have discovered conflicting evidence that shape information is also encoded in the dorsal pathway, and spatial information (including angle of gaze) is also encoded in the ventral pathway. For example, in physiological studies, shape selectivities, shape memory, and even shape remapping have been found in area lateral intraparietal cortex (LIP) in the dorsal pathway (Subramanian & Colby, 2014; Sereno & Amador, 2006; Sereno & Maunsell, 1998). In human fMRI studies, shape activities have also been found in the dorsal pathway (Zachariou, Klatzky, & Behrmann, 2014; Konen & Kastner, 2008). In addition, spatial selectivities have been found in the ventral pathway in physiology studies (Lehky, Sereno, & Sereno, 2016; Sereno, Sereno, & Lehky, 2014; Sereno & Lehky, 2011) as well as human fMRI studies (Kay, Weiner, & Grill-Spector, 2015; Schwarzlose, Swisher, Dang, & Kanwisher, 2008). These findings showing shape and spatial properties in both pathways do not rule out the idea that object recognition is the goal of the ventral visual pathway and spatial processing is the goal of the dorsal visual pathway, but suggest that our understanding of the segregation of shape and spatial properties in these pathways in the brain is still incomplete and demand explanation. In addition, because of various limitations of different approaches and techniques (e.g., anatomical, physiological, imaging, or neuropsychological), it is difficult to know whether shape and spatial information are independently encoded in each pathway or whether shape and spatial information can propagate to different streams through multiple direct interstream or more indirect (e.g., prefrontal, thalamic, or subcortical) communications. Therefore, artificial neural networks with controlled and manipulated connectivity can be helpful and critical to help investigate these issues. For example, Hong, Yamins, Majaj, and DiCarlo (2016) found in electrophysiology experiments that explicit spatial information increased along the ventral pathway, and then confirmed these findings by making explicit assumptions and doing simulations using artificial neural networks.

## SIMILAR PROPERTIES IN THE TWO CORTICAL VISUAL PATHWAYS

A first controversial issue is whether the reports of mixed properties, that is, object properties in dorsal and spatial properties in ventral, are similarly represented possibly because of interstream communication (Milner, 2017; Sayala, Sala, & Courtney, 2006) or whether object and space are independently processed and differently retained by each pathway (Sereno, Lehky, & Sereno, 2020).

Konen and Kastner (2008) reported that basic object information related to shape, size, and viewpoint was represented similarly in ventral and dorsal visual pathways, concluding that there were two similar and hierarchically organized pathways in ventral and dorsal cortex for object information. Likewise, although the dorsal visual pathway was believed to be responsible for processing spatial information (Mishkin et al., 1983), spatial selectivities related to an object's retinal location and angle of gaze (eye position) have also been reported in the ventral visual pathway (Sereno et al., 2014; Bremmer, 2000; Lueschow, Miller, & Desimone, 1994).

Anatomical interconnections between temporal and parietal visual areas and among prefrontal cortical regions have been previously documented (e.g., Webster, Bachevalier, & Ungerleider, 1994). Some functional studies suggested that these interconnections between the ventral and dorsal pathways may lead to spatial information in the ventral pathway and the shape information in the dorsal pathway. For example, activations found in ventral areas during a spatial task were suggested to possibly reflect inputs from dorsal areas (Sayala et al., 2006). However, Sala and Courtney (2007) suggested that these interstream connections may specifically function in a sort of competitive selection process. Nevertheless, according to Milner (2017), studies conducted with D. F. (a patient suffering from visual form agnosia because of ventral pathway damage) suggest that there is both ventral-to-dorsal and dorsal-to-ventral traffic (communications) between the two pathways. Patient D. F. had difficulty recognizing objects. When D. F. was asked to post (put) a T-shaped object into a T-shaped aperture, they found that although she was able to use the orientation of one edge to control her posting behavior, she was not able to combine two visual orientations to form a composite shape to guide her posting behavior. Therefore, a healthy person's ability to control their posting behavior based on the combination of multiple orientations may suggest that the shape information in the ventral-to-dorsal traffic is important for this task. Furthermore, when D. F. was asked to pick up one specific object from a display with a square and a rectangular block, they found that although she was not able to discriminate the two objects verbally, she was able to use the aperture of her grasp to help her reach the correct object. They argue that these findings suggest that the spatial information in the dorsal-to-ventral traffic could help facilitate object discrimination.

## DIFFERENCES IN SHAPE AND SPATIAL PROPERTIES IN THE TWO CORTICAL VISUAL PATHWAYS

In contrast to the idea that object and spatial properties are represented similarly across streams, Lehky and Sereno (2007) argued, based on multidimensional scaling analysis (MDS) of monkey neurophysiological data, that shape representations in ventral and dorsal cortical areas were different (i.e., not just scaled versions of each other) and instead showed significant differences in object discrimination and categorization. Specifically, although shape selective neurons were found in both pathways, Lehky and Sereno (2007) showed that neurons recorded in anterior inferotemporal cortex (AIT) in the ventral pathway, analyzed either at a single-cell or neural-population level, showed a greater capacity to make finer shape discriminations than neurons recorded in LIP in the dorsal pathway.

Furthermore, based on additional work, they suggested that the spatial representations in the ventral and dorsal pathways were also distinct. Namely, Sereno and colleagues (Sereno et al., 2014; Sereno & Lehky, 2011) recorded neural activities from AIT in the ventral pathway and LIP in the dorsal pathway of awake behaving monkeys when a stimulus was presented at different locations within the visual field. They found cells that were spatially selective for retinal and eye position in both AIT and LIP, despite having large receptive fields. However, they argued that the spatial representations in ventral and dorsal streams were distinct: Although populations of spatial selective cells in LIP could almost perfectly localize the spatial locations of a stimulus (accurate or metric spatial representation), populations of spatial selective cells in AIT showed a less accurate and only topologically correct (e.g., “next to” or “above”) representation of space (categorical spatial representation).

**Figure 1.**
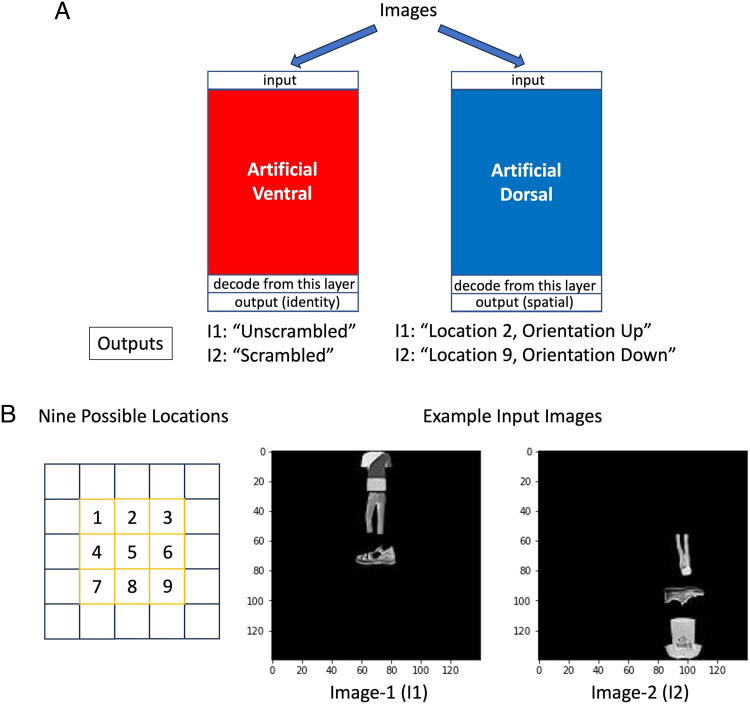
(A) Diagram of the artificial ventral and dorsal pathways. The two pathways were independently trained to determine identity (unscrambled or scrambled) and spatial information (orientation and location) of objects. (B) Possible locations and example input images. Each object can be at one of the nine possible locations (defined by the location of the part at the center). Each object consists of three parts: a t-shirt, a pant, and a shoe (part images from Xiao, Rasul, & Vollgraf, 2017). They can have orientations up, left, right, down.

Additional evidence that object and space are independently processed and differently retained by each pathway comes from computational approaches using artificial neural networks. In previous computational modeling studies using artificial neural networks, we demonstrated that shape and spatial information can be separately and independently constructed in each pathway (Han & Sereno, 2022). In this study, two convolutional neural networks (see Figure 1A) were independently trained to either recognize the identities of objects (for modeling the ventral pathway) or to determine the orientations and locations of objects (for modeling the dorsal pathway) in the input images (see Figure 1B). Each input image had a single object randomly placed at one of the nine possible locations on a black background (see Figure 1B). Each object consisted of three parts: a t-shirt, a pant, and a shoe. Objects could be presented unscrambled (the three parts are in the order of a normal person, t-shirt, pant, shoe from top to bottom) or scrambled (the three parts are in any other orders, e.g., pant, shoe, t-shirt). Each object (and their parts; all parts always had same orientation) could have four possible orientations: up, down, left, and right. Examples of the input images are shown in Figure 1B. Either nonlinear decoders (multilayer perceptrons) or linear decoders (support vector machines with a linear kernel) were used to decode information from the second to last layer of the artificial neural networks (see Figure 1A). For both methods, we found that when these artificial ventral and dorsal pathways were independently trained, it was possible to decode spatial information (orientation and location) from a ventral pathway that was only trained to recognize objects and, likewise, to decode object identity (shape) information from a dorsal pathway that was only trained to determine orientations and locations of objects. Most importantly, we showed that these artificial ventral and dorsal pathways not only actively retained both shape and spatial information but also that they retained different kinds of shape and spatial information. The artificial ventral pathway maintained more information about orientation than location, and the artificial dorsal pathway maintained more information about the identity of the whole object (unscrambled or scrambled) than the identity of a part of the object (whether the shoe was a sandal or a closed shoe). Han and Sereno (2022) argued that orientation rather than location information might be retained by the artificial ventral pathway because orientation information is more relevant to the trained task goal of the ventral pathway (identity), whereas the identity of the whole object (requiring spatial relations of parts) is more relevant to the trained task goal of the artificial dorsal pathway (spatial information). Therefore, these simulations suggest that shape and spatial information retained by the two cortical pathways may be dependent on the different task goals. Although the artificial neural network pathways were separate and independent, different kinds of shape and spatial information were retained in both pathways. These simulation findings demonstrate that it is not necessary for the ventral pathway to get spatial information from the dorsal pathway and for the dorsal pathway to get shape information from the ventral pathway. That is, simulations show that the shape and spatial information retained in the two pathways are mostly independent. In addition, based on their fMRI studies and simulations using deep neural networks, Graumann, Ciuffi, Dwivedi, Roig, and Cichy (2022) also found representations of object location along the ventral visual pathway. Furthermore, they found that the amount of location representations increased along the ventral pathway when visual images had more complex backgrounds. This finding may be because when the background is more complex, location information is more important for the ventral pathway to recognize objects (Graumann et al., 2022).

## WHY DOES THE BRAIN HAVE SEGREGATED VISUAL PATHWAYS?

**Figure 2.**
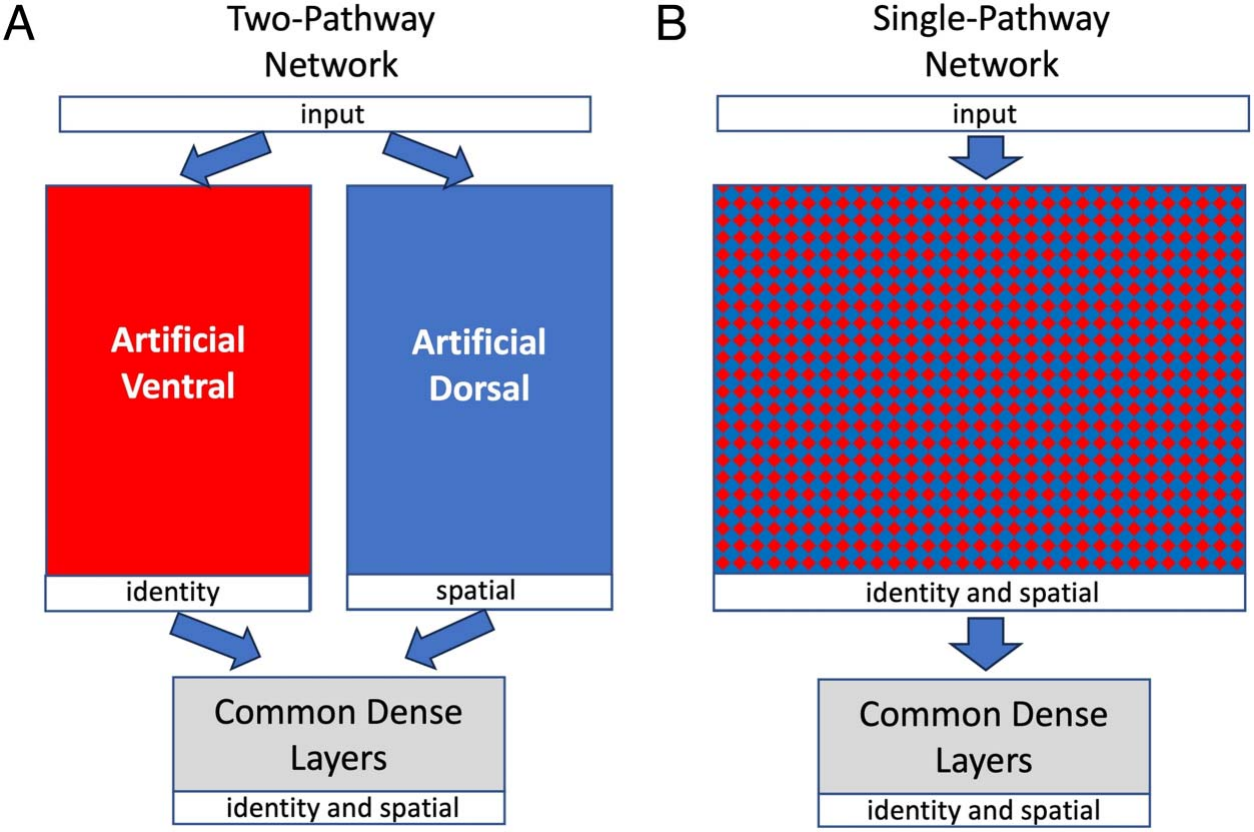
(A) Diagram of the two-pathway network. (B) Diagram of the single-pathway network. The two kinds of networks have the same number of layers and the same size in each layer (e.g., if there are 100 neurons in a layer in each pathway in the two-pathway network, then there are 200 neurons in the corresponding layer in the single-pathway network).

If ventral and dorsal pathways each retain and process both shape and space information, then why would the brain keep two segregated pathways instead of merging them into one pathway? Rueckl, Cave, and Kosslyn (1989) conducted a series of simulations using simple multilayer perceptrons to investigate the computational advantage of using two segregated pathways. They found that when there are enough computational resources in both pathways, two-pathway networks had higher performance than one-pathway networks because two-pathway networks were able to develop more efficient internal neural representations that could represent both shape and space. Instead of using simple multilayer perceptrons and simple shapes, we conducted simulations using modern convolutional neural networks and more complex object images (Han & Sereno, 2023b). In this study, each object could be either a t-shirt, a pant, or a shoe and we trained the artificial neural networks to determine the identity and spatial information of each object, when there were multiple objects in each input image. As before, we trained a separate identity network to determine the identities of the objects (t-shirt, pant, or shoe) and a second spatial network to determine the locations of the objects (nine possible locations; see Figure 2). After training, the weights in the identity and location networks were fixed and the last output layer from the two networks were removed. The information processed by the two independent pathways was then sent from the two pathways' second to last layers to common dense layers to combine them together so that the full network could be trained to simultaneously determine the identities and locations of the objects. The two pathways had already been trained, and the weights in the two pathways were fixed when the common dense layers were trained. We found that two-pathway neural networks consistently outperformed single-pathway neural networks (Han & Sereno, 2023b). Specifically, two-pathway networks were more efficient to train (required less training time) and could achieve higher testing accuracy after training. In addition, the advantage of using two-pathway networks increased when the number of objects in the input image increased (Han & Sereno, 2023b). These computational simulation results suggest that it may be computationally advantageous for the brain to maintain two segregated cortical visual pathways. Interestingly, a similar modular (as opposed to single) system has also been put forward recently as an effective solution to reduce the energy consumption of deep neural networks on small computers with limited hardware resources (Goel et al., 2020).

Although the computational simulation studies discussed above cannot exclude the possibility that the ventral pathway gets spatial information from the dorsal pathway and the dorsal pathway gets shape information from the ventral pathway, our simulation results demonstrate that even when networks are trained on a single task, shape and spatial information are independently retained and processed by each pathway.

How is it possible that several studies have demonstrated that artificial neural networks with two pathways can achieve better performance than artificial neural networks with a single pathway (Han & Sereno, 2023b; Rueckl et al., 1989)? One possible reason is that neural networks with two independent pathways have fewer parameters (connection weights) than neural networks with a single pathway, assuming that the number of layers and the number of neurons in each layer of the single- and two-pathway networks are held constant. As a result, two-pathway neural networks are easier to train and can achieve higher testing accuracy with a limited number of training samples. If there are many interconnections between the two pathways, then the number of parameters would increase and reduce network efficiency. Therefore, although there may be some communications between the two cortical visual pathways, it may be computationally advantageous that the shape and spatial information in the ventral and dorsal pathway should be independently retained and processed separately in each pathway (for its own goal).

If shape and spatial information are retained and processed separately in each pathway, then why are there interconnections (e.g., Webster et al., 1994)? We speculate that processing the information independently and differently provides a computational advantage but does not preclude that the information from the two pathways could be combined together to form an integrated representation of the world. Indeed, Sala and Courtney (2007) suggested that the interstream communications between the dorsal and ventral pathways may be used to implement “biased-competition” like interactions so that the brain can combine shape and space information. They found that the activation in the ventral stream during a spatial working memory task was above a resting baseline but lower than the activation of those ventral areas during an object identity working memory task. Similarly, the activation in the dorsal stream during an object identity working memory task was above a resting baseline but lower than the activation of those dorsal areas during a spatial working memory task. They also found that asking the participants to remember both the identities and locations of the objects resulted in an intermediate level of activation of both ventral and dorsal streams. The findings reported by Milner (2017) also suggest that the interstream communications could help people guide their actions and facilitate object discrimination. In our models (Han & Sereno, 2022, 2023b), the successful combining of information from the two segregated pathways using the common dense layers could be related to interstream communications, perhaps occurring in prefrontal cortical areas, and used to explain the seeming evidence of interstream communications reported by Milner (2017).

## IS IT ALWAYS BETTER TO HAVE SEGREGATED PATHWAYS?

Although much work briefly reviewed above has suggested a basic division of higher visual processing into a ventral pathway and a dorsal pathway, more recent work suggest there are likely multiple substreams within these general divisions (Taubert, Ritchie, Ungerleider, & Baker, 2022; Pitcher & Ungerleider, 2021; Kravitz, Saleem, Baker, Ungerleider, & Mishkin, 2013; Aflalo & Graziano, 2011; Kravitz, Saleem, Baker, & Mishkin, 2011). Furthermore, even within the first visual area, it has long been recognized that processing of various visual features themselves (e.g., color, form, depth, motion) occurs in separate functionally segregated streams (Livingstone & Hubel, 1988). Thus, given that previous work suggests that it was computationally advantageous to use two-pathway neural networks to process object identity and location information independently when there were multiple objects in the image (Han & Sereno, 2023b), does the brain always use segregated pathways to process different visual attributes independently?

Recent research has found that brain-like functional specialization could emerge spontaneously in a single-pathway convolutional neural network when it was trained to recognize faces and objects (Dobs, Martinez, Kell, & Kanwisher, 2022). Therefore, when processing different kinds of information, functional segregation may arise within a single pathway. We conducted some additional simulations with convolutional neural networks to test whether two-pathway networks outperform single-pathway networks, when any two out of the following four attributes are needed to be determined by the network: object identity, luminance level, orientation, and location (Han & Sereno, 2023c). According to preliminary results, it is always computationally advantageous to use neural networks with segregated pathways to process different visual attributes independently because two-pathway networks were able to achieve significantly higher accuracy than one-pathway networks. One difference between our studies (Han & Sereno, 2023c) and the studies conducted by Dobs and colleagues (2022) is that we trained neural networks on different goals (object identity, luminance, orientation, location) whereas Dobs and colleagues (2022) trained neural networks on a single goal (object identity) with two different categories of objects (recognizing faces and recognizing non-face objects). It may be necessary for the neural network to have segregated pathways to discriminate information about multiple different attributes of objects, but if the network has a single goal (determining object identity) it may be sufficient to have a single or combined pathway. That is, if the brain needs to discriminate within each of these different visual attributes, then multiple subpathways within each of the two main cortical visual pathways may be required. However, these are speculations that require further computational and physiological investigations.

## THE BINDING PROBLEM

Another important question, often labeled as the binding problem, is how the brain binds information together from multiple segregated pathways to form an integrated representation of the world. The binding problem is especially difficult when there are multiple objects in the visual image. According to Gref, van Steenkiste, and Schmidhuber (2020), an important reason that artificial neural networks still fall short of human-level generalization is because they are not able to dynamically and flexibly bind information that is distributed throughout the network.

Both Han and Sereno (2022) and Rueckl and colleagues (1989) trained two-pathway networks using input images with a single shape or object in each image. The binding problem is trivial in this case because it is easy for the network to integrate different attributes together for a single object. However, when there are multiple objects in each input image, the binding problem becomes more difficult. Difficulty increases exponentially with the number of objects (Han & Sereno, 2022).

Prior physiological work demonstrated that the ventral pathway retains and processes both shape and spatial information, and that the spatial information retained in the ventral pathway is topological and not accurate (Sereno et al., 2014; Sereno & Lehky, 2011). In both Sereno and colleagues (2014) and Sereno and Lehky (2011), the authors demonstrate using a MDS/Procrustes analysis of neural populations that AIT in the ventral pathway had large distortions in the representation of space using three different methods. Namely, the distortion was obvious from (1) direct examination of the MDS plots; (2) from the calculated distortion (stress values) between physical space and the spatial geometry recovered from population activity; and (3) from the eigenvalues of the MDS (independent measure from stress). Hence, Han and Sereno (2023b) proposed a computational approach to constraining the binding problem using the topological spatial information in the ventral pathway. The topological spatial representations are not accurate because they are not representing the exact spatial locations of objects. However, they are precise because the topological representations are reliable. Han and Sereno (2023b) thus trained the artificial ventral pathway to determine objects' identities and reported the identities in a consistent (although arbitrary) order based on the relative locations of objects (e.g., report identities of the objects at the top left first, and identities of the objects at the bottom right last). As a result, the ventral pathway retained both the identity information and the relative location information of objects. Then, they combined the two pathways (artificial ventral and dorsal) and processed the same information with common dense layers to train the network to determine the identities and locations of multiple objects in the images. In this way, the relative location information in the ventral pathway and the absolute location information in the dorsal pathway could be combined to constrain the binding problem. By using a relative location map in the ventral pathway, the binding problem was effectively constrained and the testing accuracy of two-pathway neural networks was improved compared with the two-pathway neural networks trained without using the relative location map (Han & Sereno, 2023b).

## PROBLEMS, LIMITATIONS, AND FUTURE DIRECTIONS

Although using a location map to constrain the binding problem was effective, it raised another question: Is it always optimal to use a location map to constrain the binding problem? The proposed solution to constrain the binding problem was spatial (based on a location map) because the relative location information in the ventral pathway and the absolute location information in the dorsal pathway was used to constrain the binding problem. However, it is also possible to use a map based on other attributes to constrain the binding problem. For example, it is possible to use an identity map instead of a location map. We could train the dorsal pathway to report the absolute locations of all objects in a consistent (although arbitrary) order that is dependent on their relative identities (e.g., first t-shirts, then pants, then shoes). As a result, the dorsal pathway would retain both absolute location information and relative identity information of objects. The relative identity information in the dorsal pathway combined with the absolute identity information in the ventral pathway may also be effective to constrain the binding problem. Likewise, when the two-pathway network needs to determine other attributes, the binding problem could be constrained using maps based on those other attributes also (such as an orientation map or luminance map). According to preliminary results reported by Han and Sereno (2023a), the location map appears to be the best choice. That is, the artificial neural networks achieved a higher testing accuracy with the tasks that required binding when using the location map compared with other maps. These findings suggest that retinotopy (a map based on retinal location) and other kinds of spatial maps or reference frames may be computationally advantageous for binding in vision. Such findings are interesting but require further investigations to determine whether the location map is optimal for binding in more elaborate and rigorous testing of varying conditions. However, it is important to note that the optimal solution for artificial neural networks may not be optimal for the brain, which has many more additional constraints and goals than even the most elaborate artificial networks. Nevertheless, using artificial neural networks to simulate and test the functional consequences of the brain's peculiar organization is a powerful conceptual approach that promises insight into how the human brain functions.

In addition, we speculate that the different internal neural representations produced by the different visual pathways in the brain might facilitate functions that are similar to self-supervised learning models. In machine learning, self-supervised learning models can be trained using supervisory signals that do not need to be provided externally by humans. Perhaps similarly, the brain can recognize different kinds of objects with much less labeled data than supervised learning models. This may be because the brain can take advantage of self-supervised learning when learning to complete visual tasks. Previous machine learning studies have used distorted versions of the input images to implement contrastive self-supervised learning (Zbontar, Jing, Misra, LeCun, & Deny, 2021; Chen, Kornblith, Norouzi, & Hinton, 2020). In these models, the artificial neural networks can be trained in the following way. The networks are asked to minimize the difference between their neural representations when the input images are the distorted versions of the same original image. The networks are asked to maximize the difference between their neural representations when the input images are the distorted versions of different original images. The distorted images are typically produced by data augmentation where the images are randomly cropped, resized, color distorted, and so forth. However, some of these data augmentation methods may not be biologically plausible. We speculate that instead of using the above data augmentation methods to manipulate the original input images, the brain might produce different internal neural representations of the same input images in different visual pathways and implement contrastive self-supervised learning using these internal neural representations. In the future, it would be interesting to investigate whether the brain is doing self-supervised learning in this way and whether this kind of self-supervised learning models can outperform other machine learning models.

## SUMMARY

The brain is an immense and complicated organ. Over hundreds of years, much work has identified the peculiar and particular structure of the brain including segregation of visual processing streams and retinotopy. Using artificial neural networks, one can explore the computational consequences of the brain's architecture uncovered by neuroscience research. Using artificial neural networks, we show here that the seemingly mixed properties of shape and space within a visual stream may be properties that are retained by that stream to successfully achieve its own goal and may not need to come via cross connections. In particular, we show that there is a computational advantage for visual stream segregation. The segregation of visual streams is important for improving the accuracy and efficiency of the visual system, even when binding the features of multiple attributes. In addition, we show that the seemingly mixed properties within a stream may be used to constrain the binding problem. Finally, we suggest there may be a special advantage for binding using a location map in vision, suggesting there may be a computational advantage for cortical retinotopy in visual areas. We speculate that the different internal neural representations produced by different visual pathways might facilitate self-supervised learning so that the brain could learn to complete visual tasks more efficiently. Together, these findings not only elucidate the functional consequences of certain aspects of primate brain organization but also offer critical insights for the field of artificial intelligence. Our findings suggest computationally more advantageous and efficient ways to achieve higher level performance on more complex problems that humans are able to solve seemingly with ease.

## Acknowledgments

We thank Elizabeth M. Frazier, Sung-Mu Lee, Daniel L. McGough, and Aditya A. Shanghavi for comments on the article.

Corresponding author: Zhixian Han, Department of Psychological Sciences, Purdue University, West Lafayette, IN 47907, or via e-mail: han594@purdue.edu.

## Data Availability Statement

The code for our studies that are mentioned in this perspective is available at https://github.com/Zhixian-Han. Additional code for our studies can be requested by contacting the first author.

## Author Contributions

Zhixian Han: Conceptualization; Writing—Original draft; Writing—Review & editing. Anne B. Sereno: Conceptualization; Funding acquisition; Writing—Original draft; Writing—Review & editing.

## Funding Information

Funding to Anne B. Sereno, Purdue University and NIH CTSI Indiana State Department of Health, grant number: 20000703.

## Diversity in Citation Practices

Retrospective analysis of the citations in every article published in this journal from 2010 to 2021 reveals a persistent pattern of gender imbalance: Although the proportions of authorship teams (categorized by estimated gender identification of first author/last author) publishing in the *Journal of Cognitive Neuroscience* (*JoCN*) during this period were M(an)/M = .407, W(oman)/M = .32, M/W = .115, and W/W = .159, the comparable proportions for the articles that these authorship teams cited were M/M = .549, W/M = .257, M/W = .109, and W/W = .085 (Postle and Fulvio, *JoCN*, 34:1, pp. 1–3). Consequently, *JoCN* encourages all authors to consider gender balance explicitly when selecting which articles to cite and gives them the opportunity to report their article's gender citation balance. The authors of this paper report its proportions of citations by gender category to be: M/M = .404; W/M = .234; M/W = .234; W/W = .128.
